# Round the Hospitals

**Published:** 1919-09-27

**Authors:** 


### ROUND THE HOSPITALS.

It is very interesting to observe that the Ministry of Health is not in favour of paying inflated salaries to probationers. At a recent meeting of the Mile End Guardians it was announced that the proposal to pay £30 a year for probationer nurses had not been sanctioned by the Ministry, which suggested that a commencing salary of £25 a year was sufficient. The proposal of the Guardians to raise the

**ROUND THE HOSPITALS—(*continued*).**

Charge Nurses' salary to £60, rising by annual increment of £5 to £75, was at the same time approved. The Chairman said that the nine candidates selected had agreed to the lower sum, and recommended that the scale laid down by the Ministry of Health be adopted. The majority of the Guardians, however, insisted on their right to pay what salary they pleased, and the engagement of any probationers has therefore come to a standstill, while the Guardians are reduced to engaging emergency nurses at £3 a week. It appears there are vacancies for about forty probationers.

No parsimony as regards nurses' salaries can reasonably be attributed to the Ministry of Health, since it has recently sanctioned the following liberal scale of salaries at the West Ham Union Infirmary, Whipps Cross: Matron, £175, rising by annual increment of £12 10s. to £200; assistant matron, £135, rising by £5 increment to £145; tutor-sister, £125-135; home sister, £110-120; office sister, £100-£110; night superintendents, £85-£95; ward sisters, £70-£90, yearly increment £2 10s.; staff-nurses, £55-£60; probationer-nurses, £20, £25, £30. The Whipps Cross Hospital, which has long enjoyed a high reputation as a training-school for nurses, is placed by this reform, which does the Guardians much credit, on a level with the best hospitals, voluntary as well as municipal.

We have pleasure in calling attention to the autumn session of lectures on tuberculosis to be given at the Brompton Hospital for Consumption every Tuesday and Friday at 8 P.M. during October, November, and December, commencing with the History of Tuberculosis by Dr. Burrell on October 21. The fee for attendance at the course is £1 1s., or for lectures, with practical work, optional examination and certificate to successful candidates £2 2s. The opportunity of gaining a thorough knowledge of all the manifestations of the disease, and the best methods of combating it, should not be missed by nurses and other workers in this extensive field. A complete syllabus of the proposed lectures will be found on page 640.

The extension of welfare work in factories and workshops has often been noted in these columns. The recent advances in this branch of medical work are well displayed in the factories of the Birmingham Small Arms Company, where each group of buildings has a surgery attached with a nursing staff consisting of a matron and seven nurses. Even during the night a nurse is in attendance. During twenty-five days of June the number of patients totalled 4,314, and they paid 7,483 visits to the surgery. Over 842 accidents occurred during this period; there were 226 eye cases, nearly 3,000 surgical and over 4,000 medical visits. A good deal of dentistry is also undertaken. Treatment is given in working hours, and is met by weekly payments. A rest room is provided for men as well as for women, many of the disabled soldiers being now employed. The full account given in the *Times Engineering Supplement* for September is of great interest to nurses drawn to welfare work. The popular notion that the welfare nurse's work is confined to binding up cut fingers will need revision in the light of these details.

The Earl and Countess of Harewood received a large body of V.A.D. workers, who have done service in local auxiliary hospitals during the war, on September 13, at Harewood House, Leeds, when Surgeon-General Sir Berkeley Moynihan gave an interesting sketch of the expansion of hospitals in that district to meet war needs. In Leeds they were asked by the War Office to provide a primary hospital for 500 beds, but in the end they had beds in primary hospitals for nearly 5,000. Without the aid of the Voluntary Aid Detachments the work of the medical services could not possibly have been carried on. They had to start now in real earnest against all forms of disabling and preventable diseases, for the great mortality inflicted upon us by the Boche was absolutely trivial as compared with the mortality from such causes as tuberculosis and measles. Reference was made to the delays which had occurred in reorganising V.A.D. work throughout the country, and more than one speaker deplored the fact that no scheme for utilising the voluntary workers had yet been formulated.

The British and Russian refugees have written to express their grateful thanks to Lady Georgina Buchanan for all she has done for them since they fled from the Bolshevist terror. They recall that they arrived ill, penniless, and in many cases despairing, to be received by her with a kindly and sympathetic welcome. Employment and a living wage were found for those who were in need, and hundreds of destitute cases were relieved. Lady Georgina's departure for the Embassy at Rome with her husband cuts short her labours for the refugees to which she and her daughter have devoted their entire time during the last twelve months. Doubtless she leaves many willing coadjutors behind to carry on the work so ably organised, for the tide of Russian refugees to our shores has by no means ebbed.

At a Conference held at Shrewsbury on the 13th inst., with the Right Hon. Thomas Richards, M.P., in the chair, it was decided to form a Welsh National Committee for Nursing. A further Conference is to be held at Cardiff on October 24 to consider a scheme, now in course of preparation by a sub-committee, to discuss and lay down general lines of policy, make provision for the training and supply of nurses and found training hostels or other institutions. The meeting was attended by representatives of the North Wales and South Wales Nursing Associations, the Welsh National Memorial Association, the Montgomeryshire and the Monmouthshire Nursing Associations, and many other societies. It is hoped to secure the co-operation of all the county and borough associations and councils, the Priory for Wales, Queen Victoria's

**ROUND THE HOSPITALS—(*continued*).**

Jubilee Institute, the Ministry of Health, all the Medical Officers of Health, the voluntary hospitals, the Insurance Committees, and last, but not least, the Order of St. John of Jerusalem with the British Red Cross Society. Thus it will be seen that a very weighty project is on foot.

---

Very bad news is coming through from Poland. A Medical Commission consisting of delegates from the United States, Great Britain, France, and Italy despatched a short time ago by the League of Red Cross Societies to investigate the typhus situation has just returned to Paris. They report that the epidemic of typhus this year has been very widespread, and that it has already resulted in great mortality. The epidemic still continues and fresh cases are constantly being introduced by refugees, prisoners of war, and other persons re-entering the country from the East. The Polish Minister of Public Health appears to be doing his utmost and is aided by the Army Medical Service, but there are lamentably too few doctors and nurses as well as a great scarcity of bedding, drugs, and essential requisites. Typhus, typhoid, and dysentery are raging, and there is some danger that these diseases for lack of proper treatment and precautions may spread far beyond the confines of Poland. There would certainly be no lack of volunteers if a relief party could be despatched, but it should be adequately manned. Small spasmodic attempts at sending out medical aid and nurses are fated to be swamped completely by the magnitude of the task.

---

The nurse training-schools are all in the throes of beginning a new year's work, and there are few in which a new spirit has not arisen. In many this session is marked by the introduction of the Sister-Tutor and the matron experiences for the first time the unexampled relief of having a trained coadjutor to take much of the strain of the teaching off her hands. It will not mean the relinquishment of the matron's own course of lectures, which are priceless, both from the ripe experience dictating them and from the opportunity they afford for bringing the nursing staff into direct relations with their chief and she with them. In other respects besides that of teaching the year opens out full of promise. Hours are almost universally shorter and most conspicuously so in the Poor-Law infirmaries which have been perhaps the greatest sinners hitherto in the matter of over-work and under-staffing. The probationer shortage still holds back many institutions from introducing projected reforms, and it is a two-edged problem, for without the reforms the probationers will not draw near, and unless they approach the reforms cannot be carried out.

---

The Governors of the Gloucestershire Royal Infirmary and Eye Institution give an interesting summary in their report of the war-work undertaken by members of the nursing staff. Three sisters and thirteen nurses have left the hospital for military service since war broke out. Miss Gibbins, Miss Beamish, and Miss M. J. Hughes received the Royal Red Cross, 2nd Class, and Miss Hughes was also mentioned in despatches. Sister M. Jennings and Miss Tud, matron of the Stratford-on-Avon Hospital, both trained at the Infirmary, were awarded the Royal Red Cross, 2nd Class, and the former also the Military Medal. The influenza outbreak was exceptionally severe last autumn. Thirty-nine cases occurred among nurses and other members of the staff, twenty-two being down with it at one time. One nurse and one ward-maid died. Nurses were fortunately available from the private staff to supplement the depleted staff in the Infirmary, and this accounts for the fact that with an additional nurse the amount received for their services underwent a decrease of £180.

---

A vigorous movement is on foot for securing the funds necessary to re-build the Royal Aberdeen Hospital for Sick Children. The estimated cost is £100,000, and of this sum about £30,000 had been subscribed before the war began. The old buildings are quite inadequate for the needs of the charity and present many drawbacks and inconveniences which seriously handicap the staff. The new building is to be amply furnished with open-air accommodation for the little patients, both medical and surgical, experience having fully justified the strong convictions of the staff in this form of treatment, even so far north as Aberdeen. Under Miss Hill the training-school for children's nurses has reached a high level of efficiency, and its expansion makes a powerful appeal to those whose children owe their lives and health to the blessings of skilled nursing.

---

The Central Midwives Board for Scotland have just issued their report for last year, and are able to record most satisfactory progress. The total enrolments for the year to March 31, 1919, are 218, and previous enrolments total 3,310. Of this number 366 have passed the examination of the Board, and the rest have been admitted either as holding recognised certificates or being in *bona-fide* practice at the passing of the Act. A number of midwives still apply for *bona-fide* enrolment, and these applications are being dealt with on their own merits. In many cases in the Highlands and Islands midwives have hardly even yet received notice of the effect of the Midwives Act on their practice. Excellent work is being done in various districts by the Medical Officers of Health in explaining the rules and procedure to be obeyed and in giving instruction to the less-educated midwives to enable them to follow out the treatment prescribed. There are now ten training institutions in Scotland, and the fact that the average pass mark at the examinations is high speaks well for their efficiency. We are asked to state that the new Secretary of the Board, Mr. Herbert George Westley, M.A., LL.B., is not a medical man.

**ROUND THE HOSPITALS**—(*continued*).

A strenuous effort has been made to get the regulations relating to the bestowal of the coveted 1914-1915 Star altered so as to make doctors and nurses employed on hospital ships eligible for it. The dangers run on account of mines and submarines by the hospital ships were continuous, and *Truth* expresses indignation that while the Star is given to naval doctors and a special decoration has been provided for the mercantile marine, the staff of hospital ships get no recognition at all. It is possible even yet that something may be done to show the nation's appreciation of what the hospital-ship doctors and nurses did for the wounded. Theirs was one of the most indispensable links in the long chain of aid which stretched out from this country to every part of the world where fighting was going on, and their courage and endurance were tested to the full.

In unveiling a brass tablet at the Leeds Poor-Law Infirmary to three members of the staff who have laid down their lives in devotion to duty, the Lady Mayoress said it was the first memorial set up in Leeds to any woman who had served in the war. Nurse Nellie Spindler, one of the three brave sisters, was killed at Abbeville by a bomb in August 1917. The other two were Nurse Elsie Pickard and Nurse Isabella Renwick, who died of influenza contracted from their patients. Leeds is justly proud of its infirmary, which is a fine well-equipped institution and training-school for nurses. Some of the wards are still in occupation by the military, but it is gradually resuming its normal activities.

The inquest held at Southport on two illegitimate babies who died at the Dr. Rimmer Memorial Nursing Home in that town, ended with a verdict of death from natural causes. Four other illegitimate babies had recently died in the home, but the proprietress, Mrs. Rimmer, was able to show from medical evidence that the cause of death was infective enteritis. The coroner expressed himself satisfied that Mrs. Rimmer was not making money out of the home, and was properly qualified for her duties. He blamed her for delaying to give notice of the death of another baby when the inquest was held on the first one. Eight of the remaining infants were removed to the fever hospital, and it was shown that the drains were in a bad condition. Without questioning the high motives of the proprietress in maintaining the home, where she was able to show that only one death had occurred in the last five years, we suggest that the case forcibly illustrates the need for closer and more continuous inspection of all homes of this description.

The matron of the Hertford and Ware Joint Hospital Board asked in her report last week for consideration of the question of the nurses' war bonus. At present the nurses get £40 a year and 2s. a week war bonus, and owing to the poor terms offered the matron can get no replies to advertisements for nurses and probationers. In similar institutions salaries offered are £45 with 15s. weekly bonus. It was decided to increase the war bonus to 5s., a measure which may possibly ease the situation for a time, but cannot be considered adequate.

A sentence of five years' penal servitude was passed at the Old Bailey on September 20 on Beatrice Hatchard, keeper of a baby-farm at Leytonstone, for the manslaughter of two children and shocking neglect of two others. Nine children were housed under terrible conditions, and the police on entering the premises found one baby dead in a perambulator.

Miss Clara D. Noyes, who has succeeded to the office of Miss Delano as Director of the Department of Nursing of the American Red Cross, has by no means entered upon a sinecure office. She was for some time an active coadjutor of Miss Delano, and previous to her Red Cross work was superintendent of nurses at St. Luke's Hospital, New Bedford, and at Bellevue, New York. Her portrait, which appears in the September number of the *American Journal of Nursing*, reveals her as a woman of singular charm and poise, apt for direction and accustomed to look below the surface of things. A request has reached the American Red Cross for the establishment of a nurse training-school in Prague under the auspices of Dr. Alice Masary, the result of this bold experiment will be followed in this country with much interest. Athens also has asked for help in setting up a training-school for nurses. At the Vladivostock Hospital Miss Dewer started a training-school for Russian nurses on April 1, the Chinese native nurses having been compelled to return to their own country. A Visiting Nurse Association has been founded at Bordeaux with American-Graduate nurses, and in all these foreign ventures the Red Cross has the assistance of an influential local Committee. It is an excellent forward policy.

### Lectures on Tuberculosis.

AUTUMN SESSION.

